# Metformin in Patients With COVID-19: A Systematic Review and Meta-Analysis

**DOI:** 10.3389/fmed.2021.704666

**Published:** 2021-08-19

**Authors:** Yin Li, Xue Yang, Peijing Yan, Tong Sun, Zhi Zeng, Sheyu Li

**Affiliations:** ^1^“Double First-Class” Construction Office, West China Hospital, Sichuan University, Chengdu, China; ^2^Department of Epidemiology and Biostatistics, West China School of Public Health and West China Fourth Hospital, Sichuan University, Chengdu, China; ^3^Department of Neurosurgery, West China Hospital, Sichuan University, Chengdu, China; ^4^Department of Cardiology, West China Hospital, Sichuan University, Chengdu, China; ^5^Department of Endocrinology and Metabolism, West China Hospital, Sichuan University, Chengdu, China; ^6^Department of Guideline and Rapid Recommendation, Chinese Evidence-Based Medicine Center, Cochrane China Center and MAGIC China Center, West China Hospital, Sichuan University, Chengdu, China

**Keywords:** metformin, COVID-19, mortality, hospitalization, intubation, deterioration

## Abstract

**Importance/Background:** The coronavirus disease (COVID-19) pandemic is a critical public health issue. Evidence has shown that metformin favorably influences COVID-19 outcomes. This study aimed to assess the benefits and risks of metformin in COVID-19 patients.

**Methods:** We searched the PubMed, Embase, Cochrane Library, and Chinese Biomedical Literature Database from inception to February 18, 2021. Observational studies assessing the association between metformin use and the outcomes of COVID-19 patients were included. The primary outcome was mortality, and the secondary outcomes included intubation, deterioration, and hospitalization. Random-effects weighted models were used to pool the specific effect sizes. Subgroup analyses were conducted by stratifying the meta-analysis by region, diabetic status, the adoption of multivariate model, age, risk of bias, and timing for adding metformin.

**Results:** We identified 28 studies with 2,910,462 participants. Meta-analysis of 19 studies showed that metformin is associated with 34% lower COVID-19 mortality [odds ratio (OR), 0.66; 95% confidence interval (CI), 0.56–0.78; *I*^2^ = 67.9%] and 27% lower hospitalization rate (pooled OR, 0.73; 95% CI, 0.53–1.00; *I*^2^ = 16.8%). However, we did not identify any subgroup effects. The meta-analysis did not identify statistically significant association between metformin and intubation and deterioration of COVID-19 (OR, 0.94; 95% CI, 0.77–1.16; *I*^2^ = 0.0% for intubation and OR, 2.04; 95% CI, 0.65–6.34; *I*^2^ = 79.4% for deterioration of COVID-19), respectively.

**Conclusions:** Metformin use among COVID-19 patients was associated with a reduced risk of mortality and hospitalization. Our findings suggest a relative benefit for metformin use in nursing home and hospitalized COVID-19 patients. However, randomized controlled trials are warranted to confirm the association between metformin use and COVID-19 outcomes.

**Study Registration:** The study was registered on the PROSPERO on Feb 23, 2021 (CRD42021238722).

## Introduction

The novel coronavirus disease (COVID-19), caused by the infection of severe acute respiratory syndrome coronavirus 2 (SARS-CoV-2), is changing the everyday life of human-being globally (1). The overall COVID-19 mortality has reached 5% worldwide, with 1/4 of patients hospitalized with COVID-19 requiring intensive care (2, 3). Novel approaches targeting COVID-19 is called in emergency (4, 5).

Type 2 diabetes is one of the most prevalent non-communicable diseases worldwide (6), affecting over 100 million adults in China (7, 8). Both elevated and fluctuated glucose levels are leading to the death and complication in people living with type 2 diabetes (9, 10). Type 2 diabetes shares common risk factors with COVID-19 fatality including age, sex, and obesity (11), and type 2 diabetes itself is one of the top risk factors for the prognosis of COVID-19 (12). Metformin is one of the most widely used anti-diabetic drugs in the past few decades (13–15). In addition to the effect of lowering blood glucose, metformin protects diabetic people from cardiovascular disease, microvascular complications of diabetes and neoplasm (16, 17).

Although a recent large cohort from England (18) and a meta-analyses (19, 20) suggested that metformin using was associated with lower mortality of COVID-19 patients, the potential benefits of metformin in patients with COVID-19 remain uncertain. The heterogeneous reporting may be attributed to different study design, health care system, study population and statistical approaches. We, therefore, conducted this systematic review and meta-analysis to explore the impact of metformin use on COVID-19 mortality and other patient-important outcomes across various populations and studies.

## Methods

### Study Registration

The study was registered on the PROSPERO on Feb 23, 2021 (CRD42021238722).

### Search Strategy

Our study followed the preferred reporting items for systematic reviews and meta-analysis (PRISMA) guidelines (21). The literature search was performed in PubMed, Embase, Cochrane Library, and Chinese Biomedical Literature Database from inception to February 18, 2021. The medical subject headings and keywords searched consisted of “COVID-19,” “SARS-CoV-2,” “coronavirus,” and “metformin.” The detailed search strategy for PubMed is shown in Supplementary Table 1. The hand-search from the references and citations supplements the relevant literature. We searched the literature using a combination of titles/abstracts and medical subject headings. Studies were included if they: (1) were designed as cohort, cross-sectional, case-control studies; (2) compared the rates of death, risk of intubation, disease deterioration, hospitalization, acute respiratory distress syndrome (ARDS), acidosis, and heart failure in COVID-19 patients with and without using metformin, and (3) reported the effect size including odds ratios (ORs), relative risks (RRs), or hazard ratios (HRs) and their 95% confidence intervals (CIs). We excluded abstracts, case reports, letters, reviews, meta-analyses, registered clinical trials not yet completed, and studies that were not English publications. Two authors (YL and TS) independently screened the titles and abstracts identified from the aforementioned databases, and then, full-text articles were read to assess eligibility. Divergences were resolved with the help of a third evaluator (ZZ).

### Data Extraction

Data were extracted according to a standardized form and included basic information (i.e., first author name, publication year, region, study design), data on participants (i.e., sample size, age, sex, diabetic status, follow-up duration), and data on outcomes [i.e., death, intubation, admission to intensive care unit (ICU), disease deterioration, length of stay, adverse events]. We contacted the corresponding authors to request any missing or unclear data and excluded the study if data was not supplied. Multivariable-adjusted ORs, RRs, or HRs and their 95% CIs were extracted from the models with full adjustment for potential confounders if more than one model was established.

### Risk of Bias Assessment

The Newcastle-Ottawa Scale was used to assess the risk of bias in the included studies (22). The risks were estimated using the following three terms: patient selection (4 items), study comparability (1 item), and outcome assessment (3 items), and the results were divided into three grades according to the total score (good, 7–9; fair, 5–6; poor, 0–4).

### Data Syntheses

By identifying a newly published large-scale study in England, we added an exploratory sensitivity analysis to test the robustness of the results. The following analyses were performed to estimate the association between metformin use and mortality among COVID-19 patients. Heterogeneity was estimated using *I*^2^ statistics. We used random-effects weighted models to pool the specific effect sizes and their 95% CIs if *I*^2^ was >50%, while the fixed-effects weighted models were used if *I*^2^ was ≤ 50%. RRs and HRs were regarded as approximates for ORs.

Exploratory subgroup analyses were conducted by stratifying the meta-analysis by region (Europe, Asia, or America), diabetic status (population with or without diabetes), the adoption of multivariate model (adopt or not), age (<60 years, ≥60 years, or not available), risk of bias (poor, fair, and good), and timing for metformin administration (before and after the diagnosis of COVID-19). We applied visual funnel plots, Egger's tests, and Begg's tests to assess for potential publication bias (23). In addition, we conducted a sensitivity analysis by excluding the study with the largest number of sample size and an influencing analysis by successively excluding one study at a time to evaluate whether the result was robust. All statistical analyses were conducted using Stata 15.0 Software (StataCorp., College Station, TX, USA), and two-tailed *P*-values < 0.05 were considered to be statistically significant.

## Results

### Study Selection and Characteristics

We identified 294 articles during the literature search and one by hand-search, and included 19 studies with 2,903,435 patients for the meta-analysis of the association between metformin use and risk of mortality (Supplementary Figure 1) (24–50). The characteristics of the included studies are presented in Table 1 and Supplementary Table 2. Of these 28 studies, two were pre-print studies. Seven of the 28 included studies were conducted in America; 14, Asia; and 7, Europe. According to the quality assessment criteria, 16 studies were of good quality; 11, fair quality; and 1 study, poor quality (Supplementary Table 3).

**Table 1:** Characteristics of included studies.

**References**	**Country**	**Region**	**Study design**	**Sample sizes M/C**	**Population with COVID-19**			**Follow-up time (day)**	**COVID-19 definition**	**Risk of bias score**
					**Proportion of diabetes**	**Age (year) mean ± SD**	**Male (*n*, %)**			
Izzi Engbeaya et al. (39)	UK	Europe	Retrospective cohort study	169/168	1.00	65.8 ± 17.5	202, 60.0%	30	SARS-CoV-2 swab-positive (RT-PCR)	5
Mirsoleymani et al. (45)	Iran	Asia	Retrospective cohort study	36/69	0.11	59.8 ± 17.2	76, 72.5%	8 to 25	Chest CT	6
Liu et al. (43)	China	Asia	Retrospective cohort study	18/46	0.33	66.0 ± 3.0	35, 54.7%	NA	SARS-CoV-2 infection (RT-PCR)	7
Jiang et al. (40)	China	Asia	Retrospective cohort study	100/228	1.00	66.1 ± 4.1	174, 53.1%	NA	WHO interim guidance and the Diagnosis and Treatment Protocol for Coronavirus Pneumonia (trial version 7) released by National Health Commission of China	8
Wang et al. (28)	UK	Europe	Retrospective cohort study	10183/10183	1.00	67.5 ± 12.5	10,623, 52.2%	NA	SARS-CoV-2 swab-positive (RT-PCR)or positive anti-body tests	9
Choi et al. (35)	South Korea	Asia	Retrospective cohort study	12/281	0.72	29.0 ± 5.0	214,73.0%	NA	SARS-CoV-2 (RT-PCR) nasal and oropharyngeal swabs-positive	7
Kim et al. (41)	South Korea	Asia	Retrospectively observational study	113/969	0.22	>18	384, 35.5%	NA	SARS-CoV-2 (RT-PCR) nasal and oropharyngeal swabs-positive	8
Orioli et al. (27)	Belgium	Europe	Retrospective study	45/23	0.89	69.0 ± 14.0	35, 48.0%	NA	SARS-CoV-2 (RT-PCR) nasal and oropharyngeal swabs-positive	6
Do et al. (38)	South Korea	Asia	Retrospective study	469/1301	1.00	60.7	1,056, 59.7%	NA	ICD-10-CM	8
Bramante et al. (24)	US	America	Retrospective cohort study	2333/3923	1.00	74.9 ± 4.2	2,954, 47.2%	NA	SARS-CoV-2 (RT-PCR), or manual chart review by UHG, or reported from thr hospital to UHG	6
Lally et al. (25)	US	America	Retrospective cohort study	127/476	0.40	75.9 ± 11.2	586, 97.2%	30	SARS-CoV-2 infection	8
Cheng et al. (34)	China	Asia	Retrospective cohort study	678/535	1.00	63.3 ± 3.5	632, 52.1%	NA	NA	8
Luo et al. (44)	China	Asia	Retrospective study	104/179	1.00	64.3 ± 3.4	156, 55.1%	NA	The diagnosis procedures of COVID-19 were referred to the Diagnosis and Treatment of Pneumonia Infected by Novel Coronavirus issued by the National Health Commission of China.	6
Li et al. (5)	China	Asia	Retrospective cohort study	37/94	1.00	66.8 ± 11.6	74, 56.5%	60	SARS-CoV-2 (RT-PCR) nasal and oropharyngeal swabs-positive	6
Lalau et al. (51)	France	Europe	Nationwide observational study	1496/953	1.00	70.9 ± 12.5	1,568, 64.0%	28	SARS-CoV-2 (RT-PCR) nasal and oropharyngeal swabs-positive	6
Crouse et al. (36)	US	America	Retrospective study	76/144	1.00	NA	272, 45.0%	NA	SARS-CoV-2 (RT-PCR) respiratory specimens-positive	7
Perez-Belmonte et al. (46)	Spain	Europe	Retrospective cohort study	1618/1048	1.00	74.9 ± 8.4	1,647, 61.9%	NA	SARS-CoV-2 (RT-PCR)	7
Bramante et al. (30)	US	America	Retrospective cohort study	NA/NA	NA	46.0 ± 28.2	2,680, 40.0%	NA	SARS-CoV-2 (RT-PCR)-positive	6
Bramante et al. (32)	US	America	Retrospective cohort study	2333/3923	1.00	70.0 ± 16.3	2,954, 47.2%	90	SARS-CoV-2 (RT-PCR), or manual chart review by UHG, or reported from the hospital to UHG	8
Bramante et al. (24)	US	America	Retrospective cohort study	676/8879	0.205	55.0 ± 16.8	4,519, 47.3%	NA	SARS-CoV-2 (RT-PCR) -positive	8
Cariou et al. (33)	France	Europe	Retrospective study	746/571	0.885	69.8 ± 13.0	855, 64.9%	NA	SARS-CoV-2 (RT-PCR) -positive and/or clinically/radiologically (i.e., as ground-glass opacity and/or crazy paving on chest computed tomography [CT] scan)	4
Nafakhi et al. (26)	Iraq	Asia	Retrospective observational study	35/157	0.349	50.2 ± 15.7	91, 47.4%	NA	SARS-CoV-2 (RT-PCR) nasopharyngeal swabs-positive	8
Yitao et al. (48)	China	Asia	Retrospective cohort study	9/248	0.058	46.0 ± 17.0	140, 54.0%	20	SARS-CoV-2 (RT-PCR) nasopharyngeal swabs-positive	6
Al Hayek et al. (29)	Saudi Arabia	Asia	Retrospective study	700/106	1.00	57.6 ± 13.9	441, 54.7%	NA	SARS-CoV-2 (RT-PCR) nasopharyngeal swabs-positive	7
Zhang et al. (49)	China	Asia	Retrospective cohort study	15/37	1.00	65.5 ± 8.70	33, 63.5%	>60	SARS-CoV-2 (RT-PCR) nasopharyngeal swabs-positive	5
Gao et al. (38)	China	Asia	Case-control study	56/54	1.00	67.9 ± 5.2	46, 41.8%	NA	Guidelines on the Diagnosis and Treatment of Pneumonia Infected by Novel Coronavirus issued by the National Health Commission of China	8
Wang et al. (47)	US	America	Retrospective cohort study	9/49	0.28	67 ± 12.5	30, 52.0%	NA	SARS-CoV-2 (RT-PCR) nasopharyngeal swabs-positive	6
Khunti et al. (18)	UK	Europe	Cohort study	1800005/2851465	1.00	67 (57–77)	1593730, 55.9%	NA	Deaths were defined as COVID-19 related if the ICD-10 codes U07.1 (COVID-19, virus identified) or U07.2 (COVID-19, virus not identified) were recorded.	9

*M, metformin group; C, control group; ICD, International Classification of Diseases; SD, standard deviation; NA, not available*.

### Metformin Use and COVID-19 Mortality

**Figure 1:**
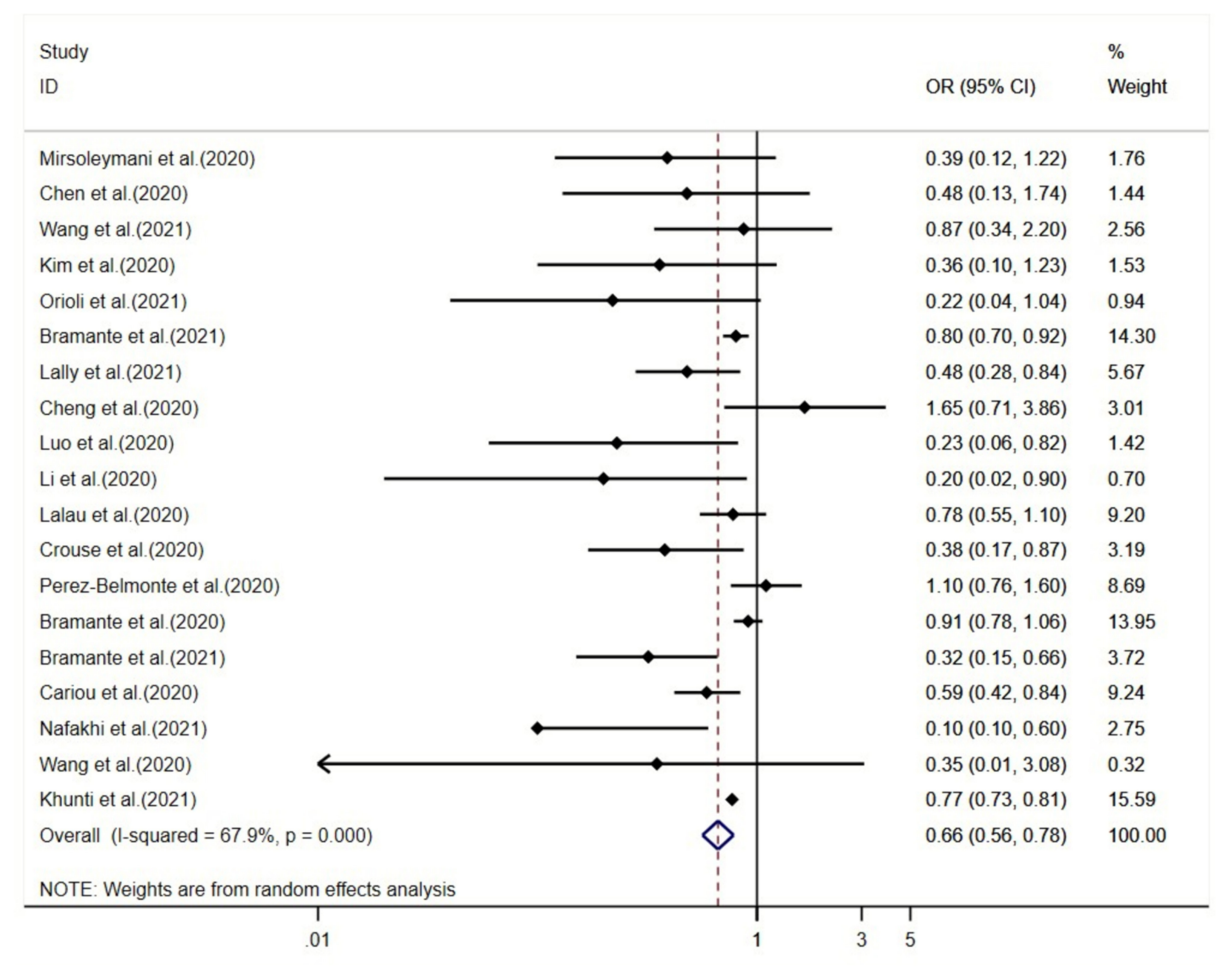
Forest plot of studies assessing association between metformin use and mortality among COVID-19 patients. CI, confidence interval; OR, odds ratio.

**Figure 2:**
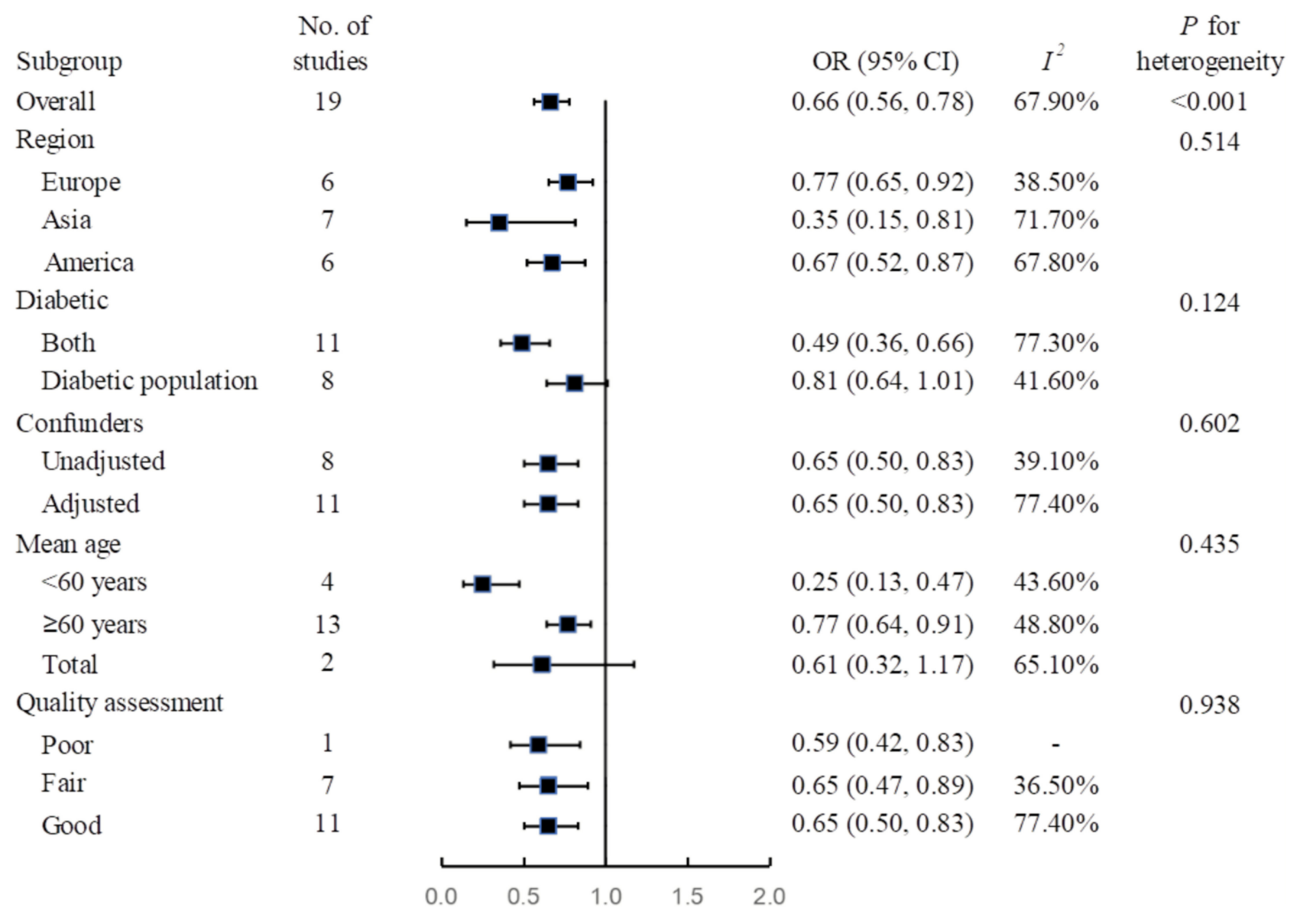
Subgroup analysis of association between metformin use and risk of mortality among COVID-19 patients. CI, confidence interval; OR, odds ratio.

The meta-analysis suggested that the use of metformin is associated with 34% lowered overall mortality (95% CI, 0.56–0.78; *I*^2^ = 67.9%) for COVID-19 patients (Figure 1). In sensitivity analysis by leaving the largest study out, the effect size shows robustness (OR, 0.60; 95% CI, 0.47–0.75; *I*^2^ = 69.7%) (Supplementary Figure 5). The subgroup analyses did not identify any credible subgroup effects (Figure 2). In addition, the influencing analysis by successively excluding one study at a time did not change the findings (Supplementary Figure 6). We did not identify significant publication bias. The funnel plot showed an approximate symmetric distribution, and the *P*-values for Egger's test and Begg's test were 0.879 and 0.270, respectively (Supplementary Figure 7).

### Metformin Use and Other COVID-19 Outcomes

To examine the association between metformin use and other outcomes, we conducted a further meta-analyses of two (33, 52), five (35, 38, 39, 41, 48, 49), and four (29–31, 47) studies estimating the association between metformin use and risk of intubation, deterioration, and hospitalization. The pooled ORs of metformin use with intubation, deterioration, and hospitalization were 0.94 (95% CI, 0.77–1.16; *I*^2^ = 0.0%), 2.04 (95% CI, 0.65–6.34; *I*^2^ = 79.4%), and 0.73 (95% CI, 0.53–1.00; *I*^2^ = 16.8%), respectively (Supplementary Figures 2–4).

In addition, it was also reported that metformin use was associated with an 82% decreased risk of ARDS (OR, 0.18; 95% CI, 0.05–0.62) (40), a 2.73-fold increased risk of acidosis (HR, 2.73; 95% CI, 1.04–7.13), and 41% decreased risk of heart failure (HR, 0.59, 95%, 0.41–0.83) (34). However, Cheng et al. did not find a significant association between metformin consumption and the risk of incident ARDS with a larger sample size (HR, 0.85; 95% CI, 0.61–1.17) (34).

The associations between metformin use and acute kidney injury, acute heart injury, and incident disseminated intravascular coagulation were also examined in this same study; however, the results were still showed no statistical significance. Moreover, metformin use did not affect the risk of in-hospital complications (46). No positive associations between metformin use and the length of ICU and hospital stays were observed in populations in Iraq with COVID-19 (26). Choi et al. reported that metformin use was associated with progression-free survival with an HR of 6.196 (95% CI, 2.58–14.91) among the Korean COVID-19 population, based on a retrospective cohort study (35), while another Korean retrospective study did not find a significant association between metformin use and COVID-19 survival (37).

## Discussion

Our systematic review and meta-analysis showed a strong association between metformin usage and reduced risk of death and hospitalization among COVID-19 patients with diabetes. This is the largest systematic review and meta-analysis on the protective effects of metformin in patients with COVID-19. Different sensitivity analyses confirmed the robustness of the results.

Metformin has been used in people with type 2 diabetes for more than half a century, proving its safety. Metformin is free of severe adverse events except for a low incidence of hyperlactacidemia in patients with advanced kidney diseases (52). Gastrointestinal adverse events are the most short-term concern for metformin affecting approximately 10% of its users (50, 53). Vitamin B12 deficiency is a potential long-term risk in metformin users, but its clinical relevance has not yet been proven (54, 55). People may choose once-daily long-acting metformin instead of metformin immediate release if they are hesitating in taking drug more than once per day (56). Reducing over 1/3 death with benefits for other health events found in the current study, the tradeoff of metformin for people with COVID-19 is clear.

Our study is in line with a recent meta-analysis including nine observational studies with 10,233 people (19), but included more people and provided information of additional outcomes. A letter to the editor also supported our findings, which reported a significant decrease in mortality with preadmission metformin use in patients with COVID-19 and diabetes (pooled OR = 0.62; 95% CI: 0.43–0.89) (20).

Mechanisms underlying the association between metformin and death and other adverse outcomes remain unclear. Recent studies demonstrated that SARS-CoV-2 uses the SARS-CoV receptor ACE2 for entry and the serine protease TMPRSS2 for S protein priming (57). The expression and stability of ACE2 is mediated by AMPK (58), which is considered as the key molecular target of metformin (59). The immunological response induced by SARS-CoV-2 infection mobilizes cytokines, mainly proinflammatory cytokines, and links to the prognosis of COVID-19 (60). Metformin reduces TNF-α to some extent in both human and animal studies (61, 62). TNF-α inhibitors are also reported to be associated with a decrease in mortality despite significant findings in a limited model (24). Thus, this pathway might partly explain the association between metformin use and mortality in COVID-19 patients.

There are some limitations that need to be noted when interpreting the results in the practice. First, the dose of metformin, strain of the virus, and the duration of metformin consumption may contribute to the heterogeneity but unavailable to be studied in this study-level analysis. Individual-level data may help explore the factors that contributes to the protective effects of metformin. Second, we did not identify any randomized trials in the systematic review and we are unable to conclude any causation of the results. The effect of metformin needs to be validated through randomized trials. Last, our study identified clear knowledge gap for outcomes of intubation and disease deterioration when current evidences are unable to support clinical interpretation. We call for further studies and analyses investigating metformin use and these two patient-important outcomes.

## Conclusion

In conclusion, metformin use was associated with reduced mortality and hospitalization in COVID-19 patients. The findings of our study indicated a relative benefit of metformin use in both nursing home residents and hospitalized patients with COVID-19. However, randomized controlled trials are warranted to confirm the association between metformin use and outcomes in COVID-19 patients.

## Data Availability Statement

The original contributions presented in the study are included in the article/Supplementary Material, further inquiries can be directed to the corresponding author/s.

## Author Contributions

YL, ZZ, and SL: conception or design. YL, XY, PY, and TS: acquisition, analysis, or interpretation. YL, XY, PY, TS, ZZ, and SL: drafting the work or revision and final approval of the manuscript. All authors contributed to the article and approved the submitted version.

## Conflict of Interest

The authors declare that the research was conducted in the absence of any commercial or financial relationships that could be construed as a potential conflict of interest.

## Publisher's Note

All claims expressed in this article are solely those of the authors and do not necessarily represent those of their affiliated organizations, or those of the publisher, the editors and the reviewers. Any product that may be evaluated in this article, or claim that may be made by its manufacturer, is not guaranteed or endorsed by the publisher.
