# Supplementary material for: Metformin in Patients With COVID-19: A Systematic Review and Meta-Analysis
Source: Front Med (Lausanne). 2021 Aug 19;8:704666. doi: 10.3389/fmed.2021.704666 (PMC8416892; doi:10.3389/fmed.2021.704666)
Supplement: Supplementary file 1 [file Data_Sheet_1.pdf]

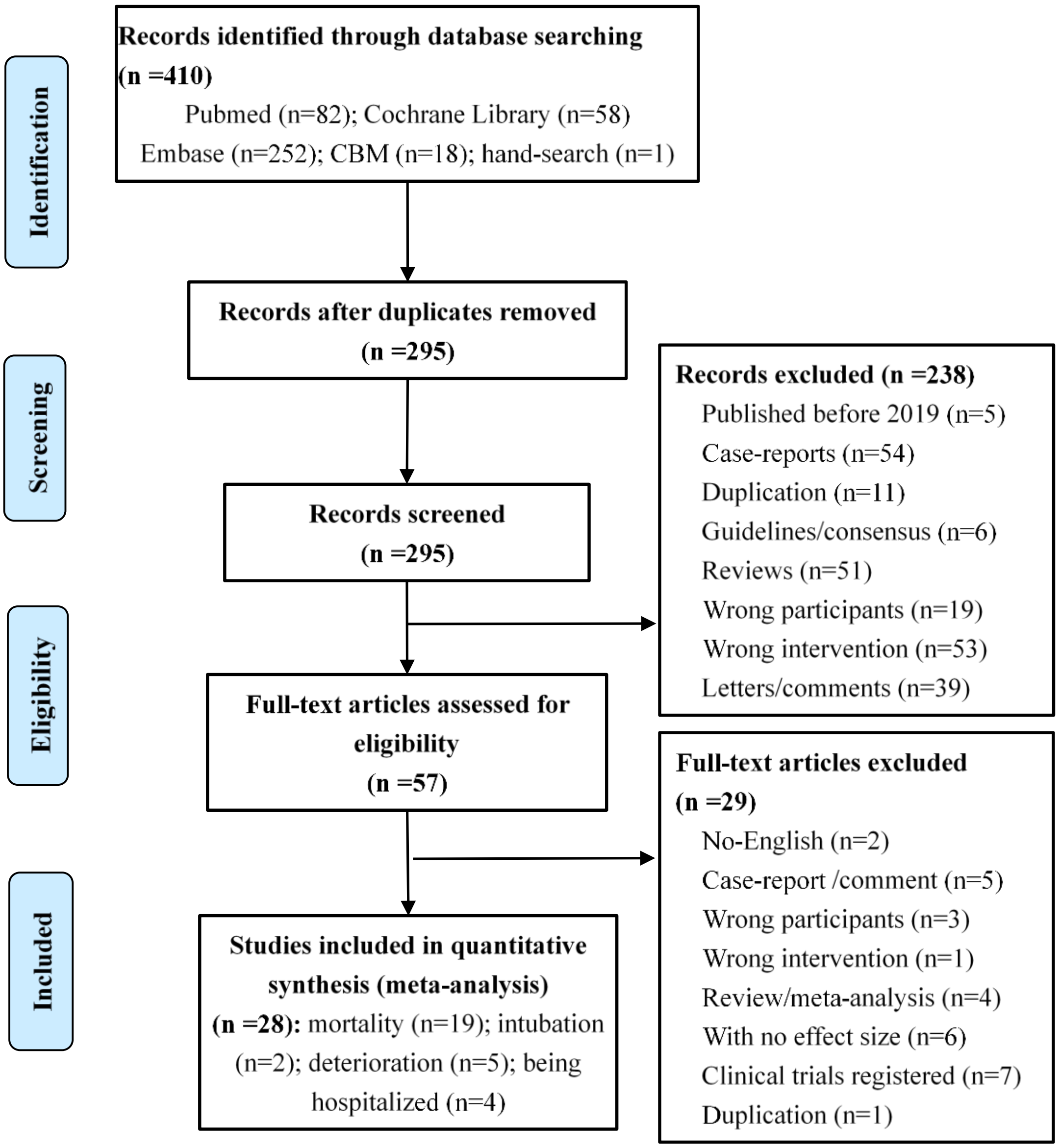

Supplementary Figure 1 Flowchart of study selection for the meta-analysis.

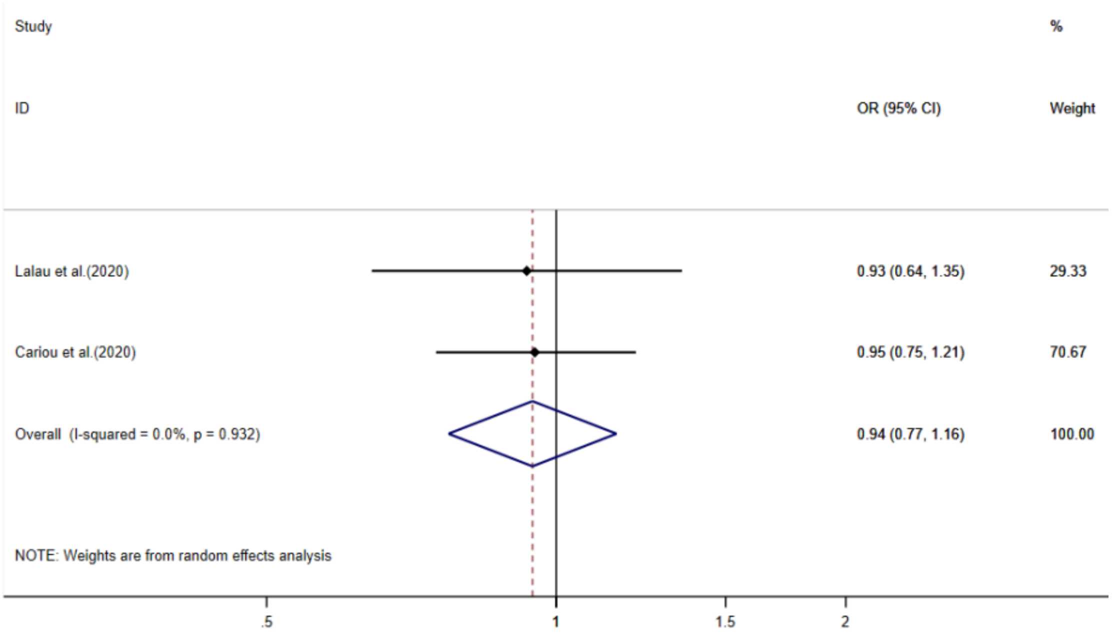

Supplementary Figure 2 Forest plot of studies assessing association between metformin use and intubation among COVID-19 patients. CI, confidence interval; OR, odds ratio.

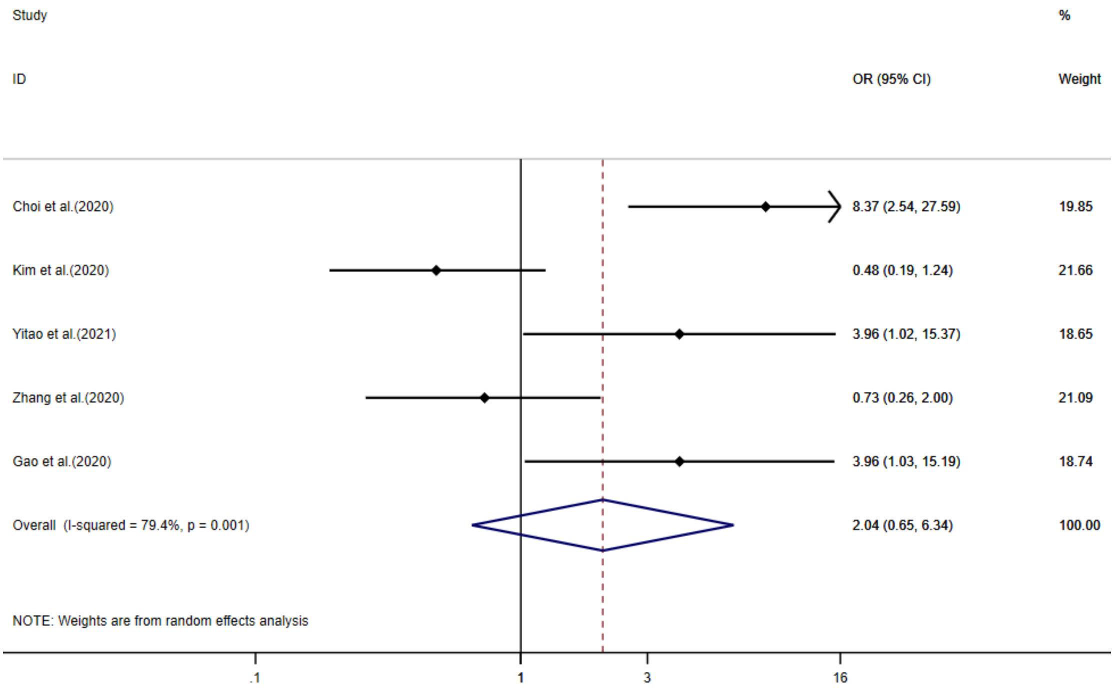

Supplementary Figure 3 Forest plot of studies assessing association between metformin use and deterioration among COVID-19 patients. CI, confidence interval; OR, odds ratio.

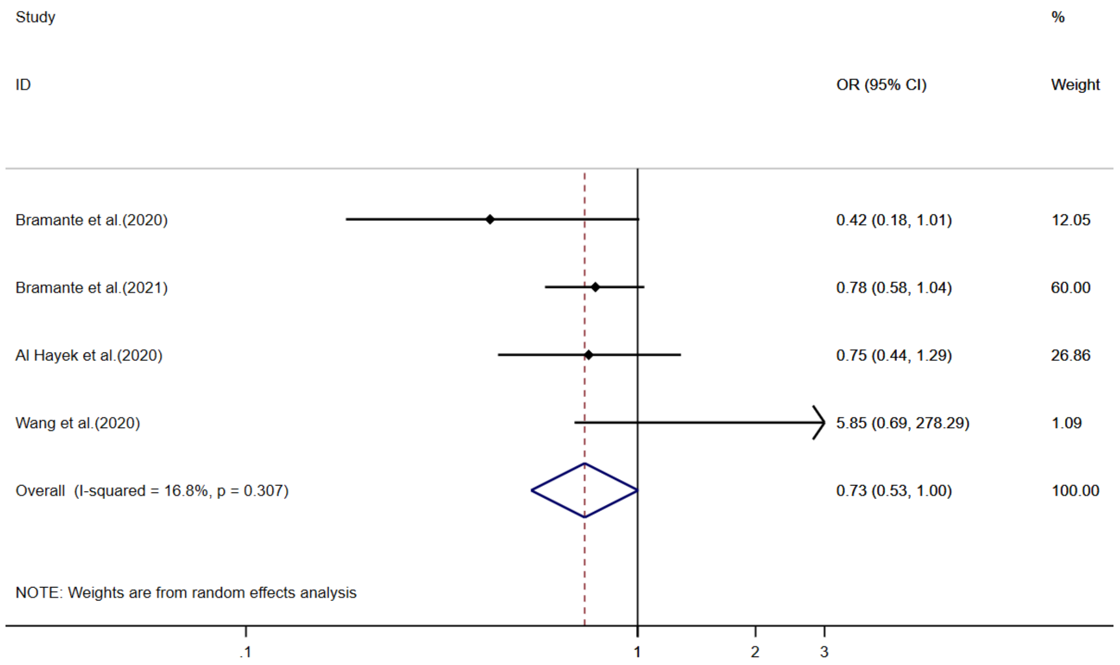

Supplementary Figure 4 Forest plot of studies assessing association between metformin use and being hospitalized among COVID-19 patients. CI, confidence interval; OR, odds ratio.

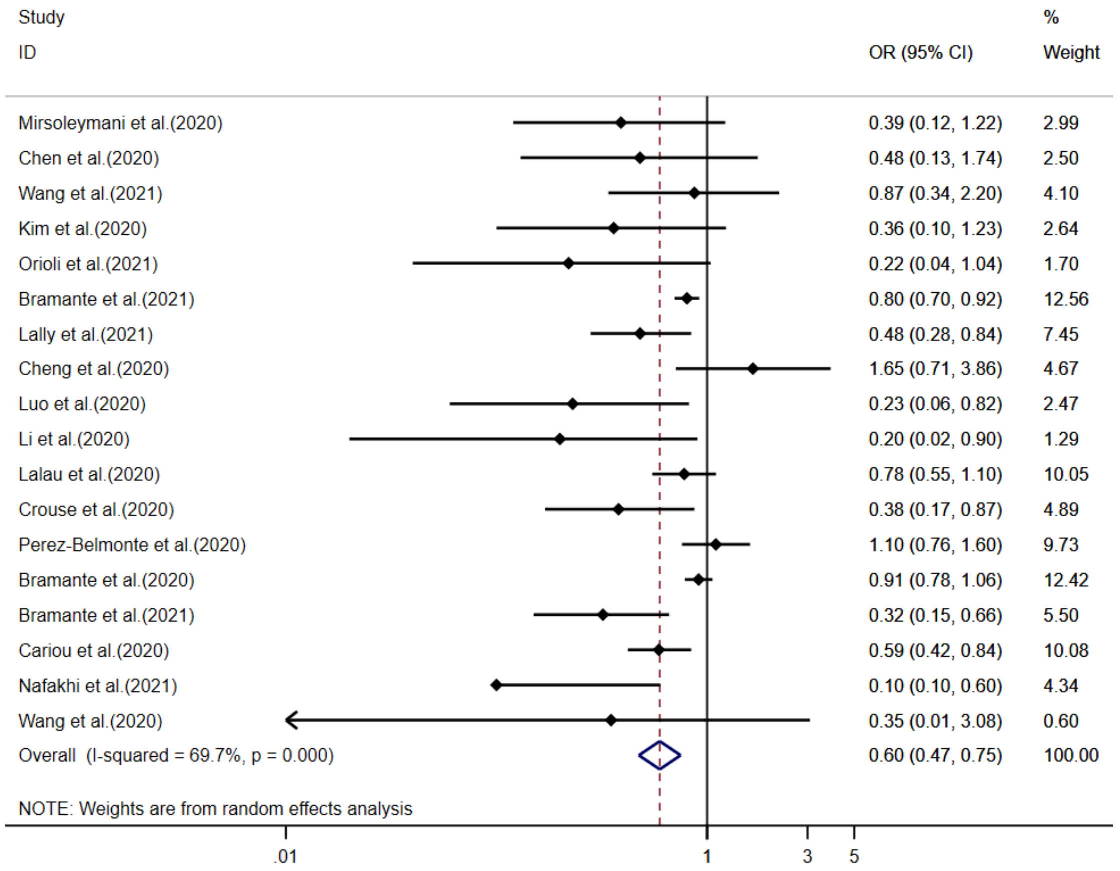

Supplementary Figure 5 Sensitivity analysis of association between metformin use and risk of COVID-19 mortality after excluded the study with the largest number of sample size. CI, confidence interval; OR, odds ratio.

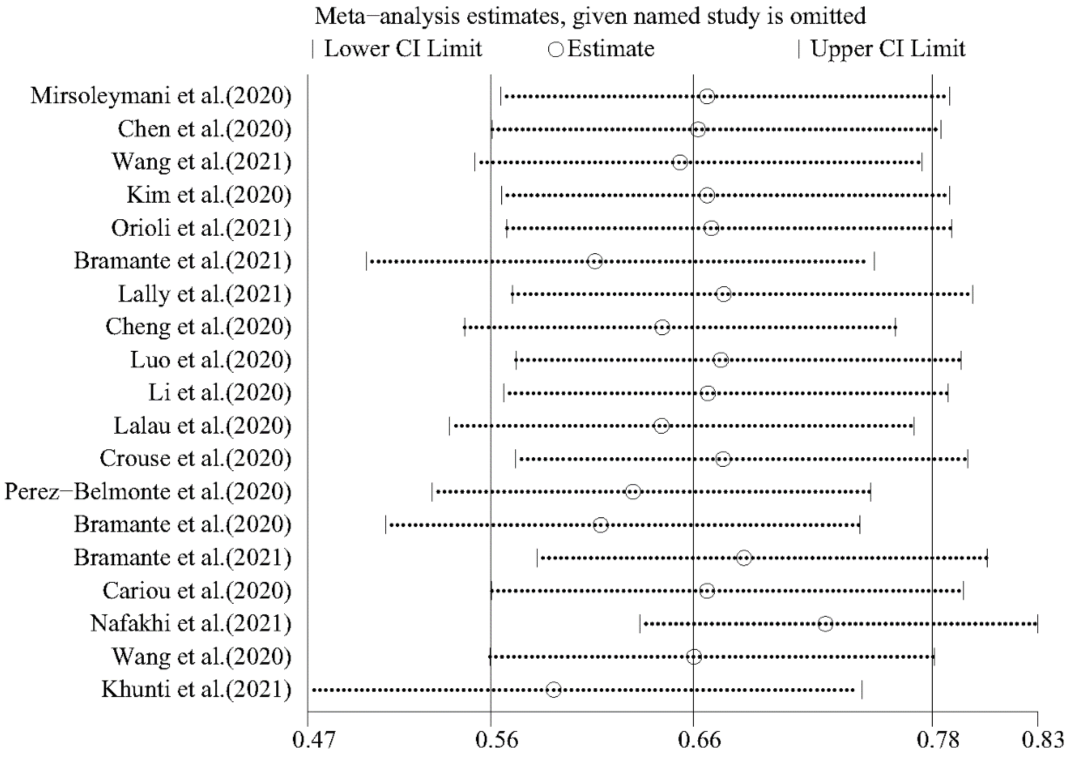

Supplementary Figure 6 Influencing analysis of association between metformin use and risk of COVID-19 mortality. CI, confidence interval; OR, odds ratio.

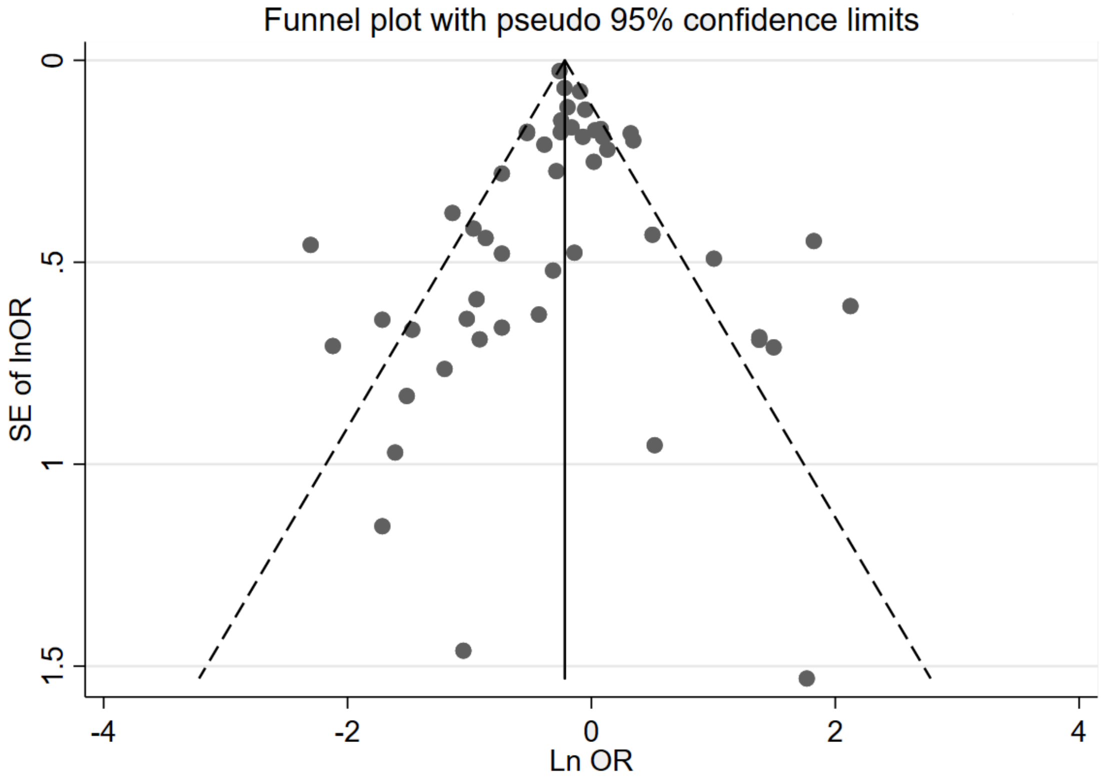

Supplementary Figure 7 Funnel plot for the studies assessing the association between metformin use and mortality among COVID-19 patients. *P* value for Egger's test = 0.879; *P* value for Begg's test = 0.270. Abbreviation: OR, odds ratio; SE, standard error.

Supplementary Table 1 Search strategy in PubMed

#1	((COVID-19 [Mesh]) OR (SARS-Cov-2 [Mesh]) OR (coronavirus [Mesh]) OR (NCov-2019))
#2	((COVID-19 [Title/Abstract]) OR (SARS-Cov-2 [Title/Abstract]) OR (coronavirus [Title/Abstract]) OR (NCov-2019 [Title/Abstract]) OR (2019-nCoV [Title/Abstract]) OR (2019 nCoV [Title/Abstract]) OR (Coronavirus Disease-19 [Title/Abstract]) OR (Coronavirus Disease 19 [Title/Abstract]) OR (Coronavirus Disease 2019 [Title/Abstract]) OR (SARS CoV 2 [Title/Abstract]) OR(2019 Novel Coronavirus [Title/Abstract]) OR (SARS Coronavirus 2 [Title/Abstract]) OR (Severe Acute Respiratory Syndrome Coronavirus 2 [Title/Abstract]))
#3	#1 OR #2
#4	(Metformin [Mesh])
#5	((Metformin [Title/Abstract]) OR (Dimethylbiguanidine [Title/Abstract] OR (Dimethylguanylguanidine [Title/Abstract]) OR (Glucophage [Title/Abstract]) OR (Metformin HCl [Title/Abstract]))
#6	#4 OR #5
#7	#3 AND #6

Supplementary Table 2 Medication, outcomes, and adjusted confounders of included studies

Author	Year	Medication			Outcomes		Adjusted potential confounders
		Metformin group	Control group	Dose	Type	Definition	
Izzi-Engbeaya et al.	2021	NA	NA	NA	Mortality/ICU	NA	None
Mirsoleymani et al..	2020	NA	NA	NA	Mortality	NA	None
Liu et al.	2020	NA	NA	NA	Mortality/ICU	NA	HbA1c at admission
Jiang et al.	2020	NA	NA	≥500 mg/d	Mortality; ARDS	NA	Age, gender, weight, FBG, severity of COVID-19, Charlson comorbidity index, glucocorticoids, DDI, LDH, CRP, duration of diabetes, metformin therapy prior to hospitalization.
Wang et al.	2021	Patients should be on a current metformin prescription (extending into the pandemic period) and co-prescribed at least one of eight other glucose-lowering agents. Patients who were on metformin monotherapy were excluded as they will be at an early disease status resulting in	Patients should not be a current or recent user of metformin and should have a prescription for at least one of the eight other glucose-lowering agents extending into the pandemic period.	NA	Mortality	Defined as individuals who died within 28 days after the diagnosis of suspected or confirmed COVID-19	Age, sex, smoking status, high alcohol consumption (alcoholism), body mass index categories, total cholesterol categories, high-density lipoprotein categories, Albumin-creatinine ratio categories, estimated glomerular filtration rate categories, HbA1c categories, atrial fibrillation, rheumatoid arthritis, hypertension, cardiovascular disease, non-alcoholic fatty liver disease or non-alcoholic steatohepatitis, prescriptions of renin angiotensin aldosterone system inhibitors, other antihypertensive drugs, lipid lowering drugs, antiplatelets, anticoagulants, diabetes complications, diabetes duration, respiratory

		immortal time bias.					disease, cancers, immunosuppressant therapies, and systemic corticosteroid use.
Choi et al.	2020	NA	NA	NA	Progression; improvement /stabilization; progression-free survival	The progression group comprised mild or moderate cases that progressed to moderate or severe cases; The improvement/stabilization group comprised mild cases that did not progress further; Progression-free survival was defined as the duration of time over which patients with COVID-19 remained stable during their hospitalization.	Age, healthcare-associated infection, ECOG performance status, asymptomatic on initial evaluation, BT at hospital admission, diastolic BP at hospital admission, PR at hospital admission, SpO2 at hospital admission, hypertension, and diabetes mellitus
Kim et al.	2020	NA	NA	NA	Mortality; progression	The necessity for the use of a high-flow nasal cannula, mechanical ventilation, CRRT, or ECMO, or admission to an ICU	None
Orioli et al.	2021	NA	NA	NA	Mortality	NA	None
Do et al.	2020	NA	NA	NA	Survival	NA	Age, gender, Charlson Comorbidity Index score,

							hypertension and group according to metformin use
Bramante et al.	2021	NA	NA	> 90 days during the year before admission to hospital	Mortality	NA	None
Lally et al.	2021	NA	NA	NA	Mortality	NA	Age, body mass index, hemoglobin A1c, estimated glomerular filtration rate, long stay (>90 days), and underlying psychoses.
Cheng et al.	2020	NA	NA	NA	Mortality; acidosis; lactic acidosis; ARDS; DIC; heart failure; acute kidney injury; acute heart injury	NA	Age, gender, comorbidities (cerebrovascular disease and coronary heart disease), blood glucose, C-reactive protein, estimated glomerular filtration rate, alanine aminotransferase, and creatinine
Luo et al.	2020	NA	NA	≥3 days	Mortality	NA	None
Li et al.	2020	NA	NA	NA	Mortality	NA	None
Lalau et al.	2020	NA	NA	NA	Mortality; IMV and Mortality in 8 days; IMV in 28 days	Tracheal intubation for mechanical ventilation	None
Crouse et al.	2020	NA	NA	NA	Mortality	NA	Age, race, sex, obesity, hypertension, diabetes, insulin in T2DM

Perez-Belmonte et al.	2020	monotherapy	other glucose-lowering drugs	NA	Mortality; Mortality/ICU/IMV; complications; long-time hospital stay	NA	None
Bramante et al.	2020	NA	NA	NA	Hospitalized	The hospital disposition indicator	None
Bramante et al.	2020	NA	NA	NA	Mortality	NA	Age, sex, pre-existing conditions, BMI, medications
Bramante et al.	2021	documented in the home medication list in the EHR within the 3 months before the positive SARS-CoV-2 PCR test	NA	NA	Mortality; hospitalized; ICU;	NA	Age, race/ethnicity, gender, English-speaking status, T2DM, BMI category, history of bariatric surgery,4 NAFLD/non-alcoholic steatohepatitis (NASH),4 coronary artery disease, heart failure, CKD; hypertension, hyper- or hypo-coagulable state, interstitial lung disease, tobacco use; and home medications: steroids; insulin, glucagon-like-peptie-1 receptor agonists 4, sulfonylureas, sodium-glucose transport protein 2 inhibitors, dipeptidyl deptidase-4 inhibitors, statins, anti-dementia medications,25 and angiotensin-converting enzyme inhibitors and angiotensin receptor blockers
Cariou et al.	2020	NA	NA	NA	Mortality; IMV in 7 days	Death within 7 days of admission	None
Nafakhi et al.	2021	NA	NA	NA	Mortality; length of ICU stays; length of hospital stays	NA	Baseline comorbidities, blood indices, and ECG markers on admission

Yitao et al.	2021	NA	NA	NA	Progression	When one or more of the following conditions occurred: 1) shortness of breath, with respiratory rates (RR) 30 times/ min; 2) arterial oxygen saturation (resting status) 93%; or 3) ratio of partial pressure of oxygen to fraction of inspiration O2 (PaO2/FiO2) 300 mmHg.	None
Al Hayek et al.	2020	NA	NA	NA	Hospitalized	Hospital admission severity criteria: i) Mild to moderate: patients who exhibited distinct upper respiratory and constitutional symptoms or patients who revealed early clinical or radiological pneumonia. ii) Severe: patients with ≥1 of the listed symptoms, respiratory rate ≥30/min, blood oxygen saturation ≤93%, partial pressure of	None

						oxygen/fraction of inspired oxygen ratio <300 or lung infiltrates >50% of the lung field from between 24-48 hours, and iii) Critical: patients who revealed ≥1 of the following symptoms such as ARDS, sepsis, altered consciousness, multi-organ failure or cytokine release syndrome.	
Zhang et al.	2020	NA	NA	NA	Progression	Acute respiratory distress syndrome, sepsis and septic shock, and those organ dysfunctions requiring admission to the ICU	None
Gao et al.	2020	NA	NA	NA	Progression	Acute respiratory distress syndrome, sepsis and septic shock, and those organ dysfunctions requiring admission to the ICU	Age, sex, blood glucose, and LDH levels
Wang et al.	2020	NA	NA	NA	Mortality; hospitalized	NA	None

Khunti et al.	2021	Prescription for metformin	No prescription for metformin	NA	COVID-19 related mortality	Deaths were defined as COVID-19 related if the ICD-10 codes U07.1 (COVID-19, virus identified) or U07.2 (COVID-19, virus not identified) were recorded.	Propensity score

ARDS, acute respiratory distress syndrome; IMV, intubation for mechanical ventilation; T2DM, Type 2 diabetes; NAFLD, non-alcoholic fatty liver disease; NA, not available.

Supplementary Table 3 Quality assessment of included studies by Newcastle-Ottawa Scale.

First author (year)	Representati-veness	Selection of non-exposed	Ascertainment of exposure	Outcome not present at start	Control for primary confounders	Control for secondary confounders	Assessment of outcome	Duration of follow-up	Adequacy of follow-up	Total
Izzi-Engbeaya et al. (2021)	1	1	1	0	0	0	0	1	1	5
Mirsoleymani et al. (2020)	1	1	1	0	0	0	1	1	1	6
Liu et al. (2020)	1	1	1	0	0	1	1	1	1	7
Jiang et al. (2020)	1	1	1	0	1	1	1	1	1	8
Wang et al. (2021)	1	1	1	0	1	1	1	1	1	9
Choi et al. (2020)	1	1	0	1	1	1	1	1	0	7
Kim et al. (2020)	1	1	1	0	1	1	1	1	1	8
Orioli et al. (2021)	1	1	1	0	0	0	1	1	1	6
Do et al. (2020)	1	1	1	0	1	1	1	1	1	8
Bramante et al. (2021)	1	1	1	0	0	0	1	1	1	6
Lally et al. (2021)	1	1	1	0	1	1	1	1	1	8
Cheng et al. (2020)	1	1	1	0	1	1	1	1	1	8
Luo et al. (2020)	1	1	1	0	0	0	1	1	1	6
Li et al. (2020)	1	1	1	0	0	0	1	1	1	6
Lalau et al. (2020)	1	1	1	0	0	0	1	1	1	6
Crouse et al. (2020)	1	1	1	0	1	0	1	1	1	7

First author (year)	Representati-veness	Selection of non-exposed	Ascertainment of exposure	Outcome not present at start	Control for primary confounders	Control for secondary confounders	Assessment of outcome	Duration of follow-up	Adequacy of follow-up	Total
Perez-Belmonte et al. (2020)	1	1	1	0	1	1	0	1	1	7
Bramante et al. (2020)	1	1	1	0	0	0	1	1	1	6
Bramante et al. (2020)	1	1	1	0	1	1	1	1	1	8
Bramante et al. (2021)	1	1	1	0	1	1	1	1	1	8
Cariou et al. (2020)	1	1	1	0	0	0	1	0	0	4
Nafakhi et al. (2021)	1	1	1	0	1	1	1	1	1	8
Yitao et al. (2021)	1	1	1	0	0	0	1	1	1	6
Al Hayek et al. (2020)	1	1	1	0	1	0	1	1	1	7
Zhang et al. (2020)	1	1	1	0	0	0	0	1	1	5
Gao et al. (2020)	1	1	1	0	1	1	1	1	1	8
Wang et al. (2020)	1	1	1	0	0	0	1	1	1	6
Khunti et al. (2021)	1	1	1	1	1	1	1	1	1	9
